# Primary Undifferentiated Pleomorphic Sarcoma of the Biceps Femoris Muscle Complicated by Hemorrhage: An Underrecognized Entity

**DOI:** 10.7759/cureus.16958

**Published:** 2021-08-06

**Authors:** Ravikanth Reddy

**Affiliations:** 1 Radiodiagnosis, St. John's Hospital, Bengaluru, IND

**Keywords:** undifferentiated pleomorphic sarcoma, biceps femoris, hemorrhage, high-resolution ultrasonography, histopathology

## Abstract

Malignant fibrous histiocytoma currently known as undifferentiated pleomorphic sarcoma is the commonest soft tissue sarcoma of mesenchymal origin. Undifferentiated pleomorphic sarcoma is commonly located in the extremities, trunk, head and neck in decreasing order of frequency. We report a case of primary undifferentiated pleomorphic sarcoma of the biceps femoris muscle in a 50-year-old male complicated by hemorrhage. Diagnostic workup included ultrasonography, magnetic resonance imaging (MRI), histopathology and positive results on immunohistochemistry especially CD-68. High-grade liposarcoma and rhabdomyosarcoma were regarded as differential diagnoses of undifferentiated pleomorphic sarcoma. Demonstration of spontaneous hemorrhage within the lesion on follow-up ultrasonography done at one month from the time of diagnosis deserves a special mention in this report. Radical excision with tumor-free margins of the biceps femoris and tendon reconstruction was undertaken. MRI at six months follow-up did not reveal tumor recurrence at the site of surgery and CT chest did not reveal metastases.

## Introduction

Malignant fibrous histiocytoma is also referred to as undifferentiated pleomorphic sarcoma, fibroxanthosarcoma or malignant fibrous xanthoma and is the most common soft tissue sarcoma usually located in the extremities with predominant musculoskeletal involvement, followed by chest, retroperitoneum, head and neck [1]. On histopathology, the classic form of undifferentiated pleomorphic sarcoma is composed of spindled fibroblast-like cells and rounded histiocyte-like cells arranged in a storiform pattern with scattered inflammatory cells and pleomorphic giant cells [2]. Less common forms of undifferentiated pleomorphic sarcoma on histopathology include either predominantly fibroblast-like cells or predominantly histiocyte like cells.

Undifferentiated pleomorphic sarcoma has a high rate of recurrence and a high likelihood of pulmonary metastasis. The malignancy typically occurs in adults between the age group 32 - 80 years with no distinct sex predilection and is usually asymptomatic until reaching advanced stage [3]. Trauma and previous surgical sites are predisposing factors for undifferentiated pleomorphic sarcoma. Local recurrence and distant metastases are commonly seen with malignant fibrous histiocytoma. Undifferentiated pleomorphic sarcoma is sometimes difficult to distinguish from high-grade sarcoma such as liposarcoma and must be included in the differential diagnosis [4].

## Case presentation

**Figure 1:**
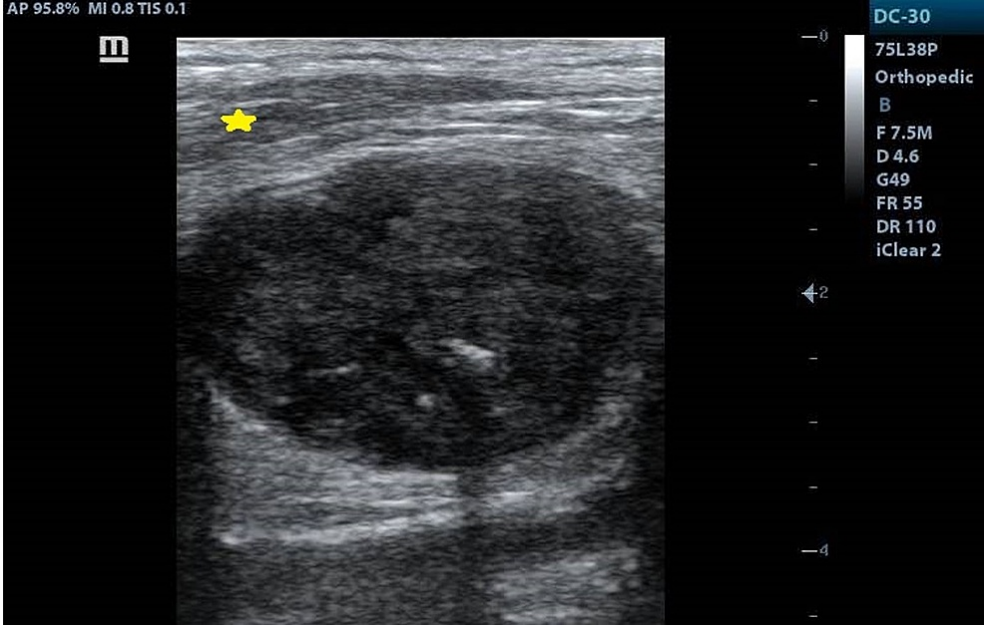
High-resolution ultrasonography image demonstrating a well-defined lobulated hypoechoic lesion with few foci of calcifications in the lower third of biceps femoris muscle of the posterior compartment in the left lower extremity. Note the semitendinosus muscle (star) located superficial to the biceps femoris.

**Figure 2:**
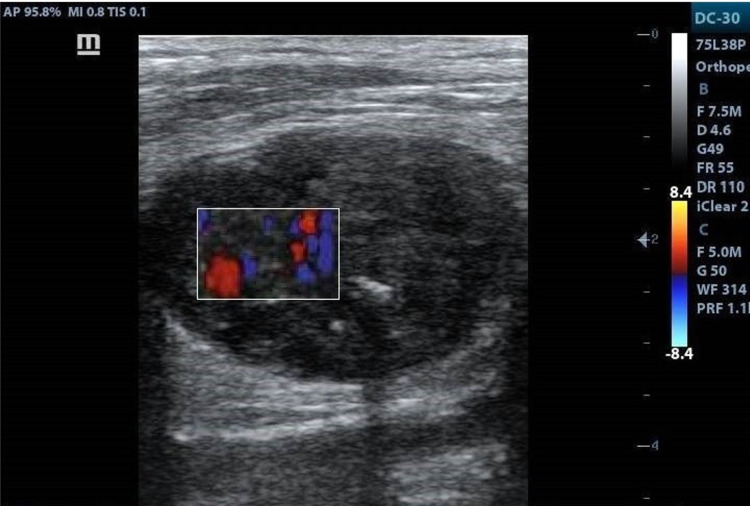
Doppler ultrasonography image demonstrating color uptake within the lobulated hypoechoic lesion consistent with features of central vascularity.

A 50-year-old male presented to the department of orthopaedic surgery with complaints of small-sized swelling in the posterior aspect of the lower thigh region in the left lower extremity for six months duration. There was no associated pain. The site of the swelling correlated with the site of trauma five years prior. At the time of initial presentation, the range of movement in the left knee joint was painful and limited. The patient was referred for ultrasonography which revealed evidence of a well-defined lobulated hypoechoic lesion measuring 3.5 x 2.9 cm with central vascularity and few foci of calcifications involving the lower third of biceps femoris muscle of the posterior compartment of the thigh in the left lower extremity (Figures 1, 2).

**Figure 3:**
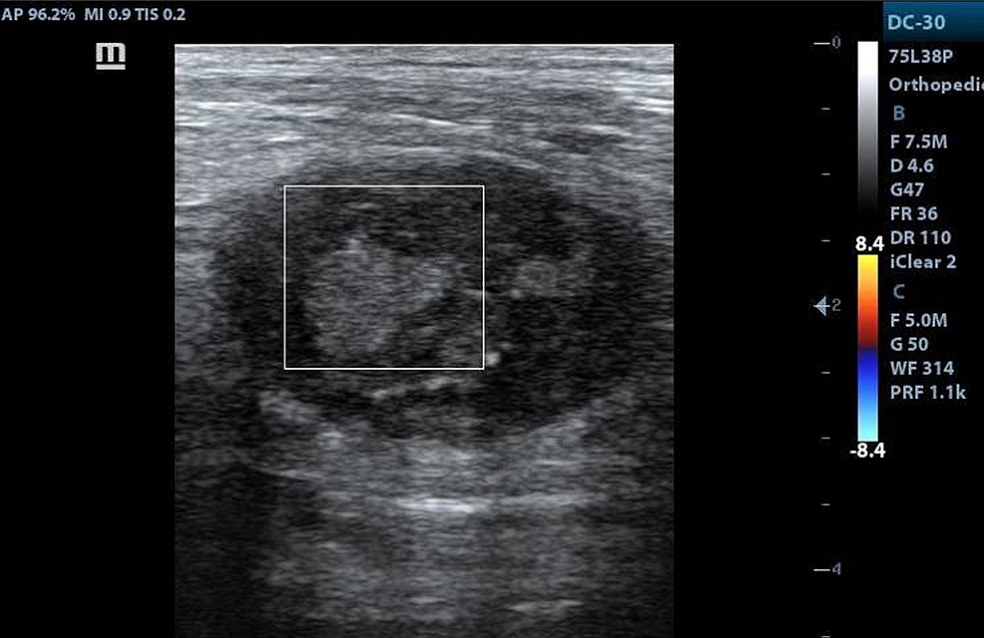
High-resolution ultrasonography image at one month follow-up demonstrating ill-defined hyperechoic areas within the lesion favored to represent spontaneous intralesional hemorrhage.

**Figure 4:**
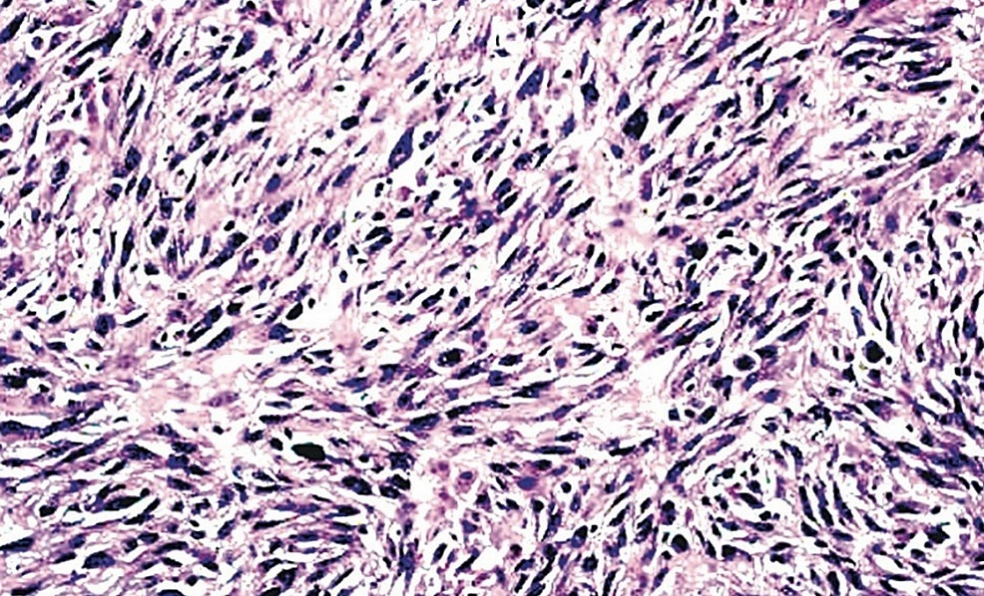
Histopathology section of the excised specimen demonstrating spindle-shaped cells arranged in a storiform pattern consistent with a diagnosis of undifferentiated pleomorphic sarcoma of the soft tissue (H and E, × 400).

Computed tomography (CT) of the chest done to rule out metastases was unremarkable. Ultrasonography-guided fine-needle aspiration cytology (FNAC) was attempted from the lesion which showed dense proliferation of spindle-shaped cells with collagenous stromal cells forming bundles in storiform patterns. A diagnosis of undifferentiated pleomorphic sarcoma was made and surgical resection was planned. Ultrasonography done at one-month follow-up demonstrated ill-defined hyperechoic areas within the lesion favored to represent spontaneous intralesional hemorrhage (Figure 3), following which radical excision with tumor-free margins of the biceps femoris and tendon reconstruction was undertaken. Surgical margins had been clear with margins as close as 2 mm. Histopathology of the resected specimen revealed a polypoidal lesion made up of spindle-shaped cell arranged in a storiform pattern around blood vessels with perivascular hyalinization consistent with features of malignant fibrous histiocytoma (Figure 4). Magnetic resonance imaging (MRI) at six months follow up did not reveal tumor recurrence at the site of surgery and CT chest did not reveal metastases. At three months follow-up, the patient had no symptoms, had achieved an excellent range of movement, and had returned to vocational and recreational activities.

The patient has given written informed consent to publish his case and clinical images.

## Discussion

Undifferentiated pleomorphic sarcoma is composed of a mixture of fibroblastic and histiocytic cells and is often reported from the skin. However, cases have been reported from deeper soft tissues of the extremities, abdomen, chest, head and neck. This case report is particularly rare as undifferentiated pleomorphic sarcoma has not been reported from the biceps femoris muscle previously. Malignant soft tissue tumors can simulate tendon tears. Tears of the proximal long head of the biceps brachii were the most common tendon tears encountered in clinical practice [5]. Diagnosis of undifferentiated pleomorphic sarcoma is difficult to make due to its varying appearances on imaging and is often confirmed after local excision of the tumor [6]. Undifferentiated pleomorphic sarcoma is a high-grade aggressive tumor often associated with a history of previous trauma and chronic infections, suggesting that malignant fibrous histiocytoma occurs secondary to chronic reactive proliferation of normal epithelium.

Undifferentiated pleomorphic sarcoma is composed of spindle-shaped cells, giant cells and round cells. Based on the distribution of above mentioned three types of cells, the findings of malignant fibrous histiocytoma on histopathology vary and are further classified into five subtypes: the classic storiform-pleomorphic type, the giant cell type, the myxoid type, the angiomatoid type and the inflammatory cell type [7]. The storiform-pleomorphic subtype is most common and constitutes 65% of total undifferentiated pleomorphic sarcomas [8]. Correlation of history, clinical findings, imaging investigations, histopathology and immunohistochemistry findings especially CD-68 is required for suspicion of undifferentiated pleomorphic sarcoma amongst lesions arising from extremities especially in the elderly population. The mainstay of treatment is radical surgical resection of the tumor with concurrent chemo­therapy and radiotherapy to reduce the risk of distant metastases and local recurrence.

Size of the primary lesion, depth of invasion, histological grade of the tumor and status of resected tumor margins are some of the prognostic factors predicting the risk of distant metastases and local recurrence in undifferentiated pleomorphic sarcoma. However, the histological grade of the tumor and the status of the resected tumor margin are the most reliable predictors of prognosis [9]. The lung is the most common site for distant metastases [10]. Long‑term follow‑up with regular chest radiographs and MRI for local staging are mandatory. The reported average five-year survival rate of patients with undifferentiated pleomorphic sarcoma is 59% - 66.7% and the local recurrence rate is 16% to 31% [11].

Ultrasonography has been able to play a key role in the management of musculoskeletal soft tissue infections, tumors and related complications. Musculoskeletal ultrasonography is an important adjunct to the other imaging modalities and sonoelastography, in particular, has been increasingly used to investigate musculoskeletal disorders and pertinent disorders [12].

Optimal management of soft-tissue sarcomas requires significant collaboration between radiologists, surgical oncologists, orthopedic specialists and radiation oncologists. Management of soft-tissue sarcomas provides a paradigm of the multidisciplinary approach for the optimization of function preservation and limb salvage. Especially, in soft tissue sarcomas affecting extremities, preserving limb function and managing limb impairment after radical local treatment represent significant challenges. Surgical management of soft-tissue sarcomas includes complete removal of the tumor and, if necessary, reconstruction of the adjacent connective tissues and neurovascular bundles. Surgical management may be considered as the primary treatment option if the tumor can be removed with adequate wide margins, without sacrificing structures critical to functional outcome. The aim of surgical procedure is to provide a wide excisional margin, which is defined as the removal of tumor and adjacent normal tissue including 2 cm of skin, fat, or muscle, and is associated with a low risk of local recurrence [13]. While wide excision with appropriate margins constitutes ideal treatment for soft tissue sarcomas, this may lead to undesirable functional outcomes, especially in extremities [14]. Vascular reconstruction of arteries commonly results in good long-term patency and extremity function. In patients with soft-tissue sarcoma of the extremities, undergoing limb-sparing surgeries, a multidisciplinary team approach is required for reconstruction and rehabilitation. 

## Conclusions

Undifferentiated pleomorphic sarcoma is the most common soft tissue sarcoma characterized by high rate of recurrence, and increased tendency for distant metastasis. Undifferentiated pleomorphic sarcoma is an aggressive high-grade soft tissue sarcoma affecting extremities most commonly in adults, and has a poor prognosis especially when distant metastases is identified on imaging. In conclusion, the imaging characteristics of undifferentiated pleomorphic sarcoma are highly variable on ultrasonography and is probably due to the degree of histological differentiation. Spontaneous intralesional hemorrhage is a related complication of undifferentiated pleomorphic sarcoma which can be reliably detected on ultrasonography as noted in this index case. Therefore, undifferentiated pleomorphic sarcoma should be included in the differential diagnosis of malignant soft tissue tumors in adult patients affecting the extremities especially and with evidence of distant metastases.
